# Gender Differences in the Effect of Grit and Resilience on Subjective Cognitive Decline Amongst Black Americans

**DOI:** 10.1002/alz70858_106231

**Published:** 2025-12-26

**Authors:** Yolanda L. Jackson, Maria F. Clark, Obinna Odo, Candidus Nwakasi, Darlingtina Esiaka

**Affiliations:** ^1^ University of Kentucky, Lexington, KY, USA; ^2^ University of Kentucky Sanders‐Brown Center on Aging, Lexington, KY, USA; ^3^ University of Kentucky College of Medicine, Lexington, KY, USA; ^4^ Miami University, Ohio, Oxford, OH, USA; ^5^ University of Connecticut, Storrs, CT, USA

## Abstract

**Background:**

Research shows that an early indicator of cognitive impairment is subjective cognitive decline (SCD). Yet, the associations of psychosocial coping mechanisms like grit and resilience with SCD, particularly in Black Americans, remain understudied. Previous research shows that grit and resilience are key coping mechanisms in Black individuals. Grit reflects perseverance toward long‐term goals, while resilience represents the ability to positively adapt and recover from adversity. Both grit and resilience have been found to increase throughout one's lifespan and are often shaped by unique cultural and social experiences. This study examined sex differences in the impact of grit and resilience on SCD.

**Methods:**

Two hundred forty‐two cognitively normal (Mean age = 44.3, SD = 8.6; 147 female) participants were recruited from CloudResearch for the study. The participants completed standardized measures assessing SCD, grit, resilience, and health and demographic characteristics. We performed linear regression analyses and reported standardized beta coefficients to describe the estimated independent effect of the predictor variables.

**Results:**

Results show that greater levels of grit are associated with higher SCD scores, with high scores indicating worse SCD. However, the relationship between grit and SCD is stronger in Black men (β = 0.704, *p* = <0.001) than in women (β = 0.528, *p* = <0.001). In contrast, higher resilience is significantly associated with lower SCD scores in Black women (β = ‐0.186, *p* = <0.002) but not in men (β = ‐0.081, *p* = <0.326).

**Conclusions:**

Our findings suggest that grit may have a worse impact on SCD in men as a coping mechanism, while resilience appears protective against SCD in Black women. These results highlight potential gender differences in how psychological traits influence the experience of cognitive health among Black Americans. Also, our study emphasizes the need to consider and further explore gender‐specific factors when addressing cognitive health in diverse populations.

